# Progress in Medicine

**Published:** 1895-09-28

**Authors:** 


### Progress in Medicine.

### DISEASES OF THE STOMACH.

**Gastric Ulcer.**—Greig, in reviewing the question of pathogenesis, considers the cause of these ulcers to be some peptic change in areas which are the seat of thrombi, or of arterial degeneration, and occasionally of injury. Ulcers are rarely, if ever, the result of emboli or hyperacidity.[1] Savelieff reports excellent results from treatment with large doses of bismuth, after Fleiner's plan. Patients take when fasting $2\frac{1}{2}$ drachms in warm water, and lie in bed afterwards for an hour with the pelvis raised so that the remedy may gravitate to the common seat of ulceration in the upper and back part of the stomach. For the rest of the day they go about their occupations, and are allowed a diet of milk, bread, rice soup, and raw meat. Food is given every two hours, and rest is enjoined after each meal. In certain chronic cases complete rest and milk diet is preferred.[2] Voiniovitch treats chronic ulcer accompanied with hyperacidity by atropine; he gives two drops of a $\frac{3}{4}$ per cent. solution of sulphate of atropine thrice daily. Papain has been largely used in cases of painful dyspepsia, with anæmia and possible ulceration, by E. G. Younger. The papain was given in one-grain doses, with an acid and sometimes with iron or morphia. Many of the patients were subjects of incipient phthisis, and nearly all showed marked improvement. As the papain retains its digestive power in alkaline as well as in acid media, its action extends to the intestines as well as to the stomach.[3] Guthrie Rankin[4] considers that in such cases the drug should be combined with iron and nux vomica and a sedative, and given generally in pill form, such as ferri sulph. papain a.a., gr. ij.; ex. cannab. ind.; ex. nuc. vom. a.a., gr. $\frac{1}{4}$; ex. rhei, gr. $\frac{1}{2}$. He also describes the results in ten cases of gastric ulcer which he treated with papain, iron, and cannabis indica in pill form, and reports very rapid recoveries. Besides the digestive properties of papain, he draws attention to its known healing power when painted over a fissure or ulceration of the tongue.[5] The relief from pain and the improvement following this treatment were so well marked that further trials are desirable. Einhorn[6] has used a spray of nitrate of silver directly to the interior of the stomach. A one or two per thousand solution is sprayed over it through a stomach tube after first washing out the organ. Intragastric galvanization is applied alternately with the spray, and a moderate mixed diet employed. The great difficulty of diagnosis of gastric ulcer was well illustrated by M. E. Lord[7] in the account of a case where violent pain and tenderness in the stomach, especially after food, vomiting of blood, and the symptoms of perforation, with collapse, were found to be due to uterine displacement, fœcal impaction, and a nasal polypus. Glyn[8] also recently discussed the diagnosis of painful digestion in hysteria from ulcer. He considered that most chlorotic patients were hysterical, and that those who were not so did not usually complain of pain after food. Still, true cases of ulcer were often complicated with both the other complaints. Henry Head pointed out that pain after food in the area supplied by the dorsal nerves from the sixth to the ninth may be caused by disturbance of any organ supplied by those nerves, thus, mitral stenosis or disease of the base of the lung may produce it. True hæmorrhage from the stomach has occurred after fatigue or emotion in neurasthenics (Mesnard),[9] and either with or replacing the catamenial flow in others. Kuttner[10] finds this much more frequent than has been supposed. When the quantity is small it can often only be detected by testing the stomach contents for the presence of blood. These cases do not yield to the treatment for gastric ulcer, though from the difficulty of disproving the presence of an ulcer in a given instance this treatment should not be omitted. In itself, this type of hæmorrhage never endangers life, but for its cure treatment of the generative system may be needed.

A rare case of peptic ulceration of the œsophagus, with perforation during life, is recorded by J. Guiteras.[11] The illness lasted seven years, there was dull pain and attacks of difficulty in swallowing, which were relieved by the passage of a bougie, but no blood was vomited. The œsophagus was found riddled with small ulcers from 4½ inches below the level of the cricoid to the cardiac orifice. Two large and perforating ones led into a cavity filled with grumous material in the mediastinum. Schneyer[12] has investigated the value of leucocytosis after meals as a means of diagnosis between simple ulcer and cancer. It was absent in all of eighteen cases of cancer, but present in most cases of ulcer. It would seem that its occurrence is strong evidence against cancer. Hæmatemesis may also be due to œsophageal varices, and it appears that these are not always dependent on portal obstruction. In a case of Friedrich's[13] it was impossible to diagnose the affection from gastric ulcer during life. Fatty degeneration of the heart and liver were found after death, and three large varices. Another point of interest was that the patient was a child of six years only.

**Cancer.**—Stewart and Boas[14] consider that the significance of lactic acid in digestion depends on the fact that in health its appearance is usually due to its introduction by bread and other baked food, and that otherwise it never occurs except in carcinoma. As it can be detected in an early stage of the latter disease, *i.e.*, at a time favourable for operation, the discovery of its presence after the stomach has been washed out is important in doubtful cases. Hammerschlag,[15] too, considers that in cancer the presence of lactic acid, the failure of the peptic glands, and the absence of leucocytosis are important symptoms taken in conjunction with the motor inadequacy of the stomach. As a remedy, sodium chlorate in daily amounts of 160 grains, divided into small doses, so that a continuous action may be kept up, has been found by Huchard to give great relief to the symptoms; but it should not be used in albuminuria, and this amount must not be exceeded. Aristol may be used in the ulcerative stage, three or four pills of a grain and a-half each are given daily.[16] Brissaud[17] also found sodium chlorate of great value in stopping the hæmatemesis, and believes it even effects a cure in some cases. Oppler[18] notices that the absence of hydrochloric acid when it occurs is due merely to the cachexia, and that the presence of lactic acid and of great quantities of thread-like bacteria is seen chiefly in those cases where the motility of the organ is impaired by cancer. Manges,[19] recognising the immense importance of early diagnosis for successful removal, relies largely upon the presence of lactic acid as an indication. The stomach having been carefully washed out, and a test meal of gruel given, he then applies Uffelman's reagent only. This is a freshly-made solution containing a few drops each of pure carbolic and ferric perchloride. The bright blue of this changes to an intense yellow if dropped into an ethereal extract of the chyme where lactic acid exists. When the presence of lactic acid and the absence of H Cl are associated, the evidence in favour of cancer is very strong, according to Manges and Boas, who add that neither sarcinæ nor sulphuretted hydrogen is found in this disease. In a discussion on the value of enlarged supra-clavicular glands as showing visceral cancer, Fernet and Girode[20] mentioned cases where tubercular disease of the viscera had produced the symptom. In infants this appears to be the usual cause, and enlargement has been found from other visceral affections. Friedenwald[21] has also reviewed the reliability of the lactic acid sign, and concludes that the acid is only produced during cancer, and then it usually appears in large quantity. The washing out of the stomach previous to the testing is important, and Uffelman's reagent can, he thinks, only be relied on where the reaction is very marked. Otherwise Boas's aldehyde reaction, which he describes fully, must be sought for. Fedora and Corselli[22] recommend betol for determining the motor power of the stomach. It is broken up only in the intestine, and appears in the urine as salicylic acid in seventy or eighty minutes after ingestion by a healthy person.

[1] Am. J. Med. Sc., April, 1895. [2] Practil, Dec., 1894. [3] Lanc. April 27, 1895. [4] Lanc., May 4, 1895. [5] Lanc., Feb. 9, 1895 [6] Therap. Gaz., Sep. 15, 1894. [7] Med. Chron., March , 1895. [8] Lanc., May 11. [9] B. M. J., March 30. [10] Med. Week, March 29. [11] Inter. Med. Mag., Nov., 1894. [12] Am. J. Med. Sc., April, 1895. [13] Practil., Jan., 1895. [14] B. M. J., March 16, 1895. [15] Med. Week. May 24. [16] Med. Rec., May [17] B. M. J., Aug. 24 1894. [18] Am. M. S. Bulletin, April 15, 1895. [19] Med. Rec., April 27, 1895. [20] Med. Week, Feb. 1, 1895. [21] N. Y. Med. J., March 23, 1895. [22] Int. Med. Mag., Sept. 1894.

